# Comparative transcriptomics of the garden dormouse hypothalamus during hibernation

**DOI:** 10.1002/2211-5463.13731

**Published:** 2023-12-18

**Authors:** Elena Haugg, Janus Borner, Gabrielle Stalder, Anna Kübber‐Heiss, Sylvain Giroud, Annika Herwig

**Affiliations:** ^1^ Institute of Neurobiology Ulm University Germany; ^2^ Sackler Institute for Comparative Genomics American Museum of Natural History New York NY USA; ^3^ Department of Interdisciplinary Life Sciences, Research Institute of Wildlife Ecology University of Veterinary Medicine Vienna Austria; ^4^ Energetics Lab, Department of Biology Northern Michigan University Marquette MI USA

**Keywords:** differential gene expression, *Eliomys quercinus*, euthermia, hibernation, metabolism, RNA‐Seq

## Abstract

Torpor or heterothermy is an energy‐saving mechanism used by endotherms to overcome harsh environmental conditions. During winter, the garden dormouse (*Eliomys quercinus*) hibernates with multiday torpor bouts and body temperatures of a few degrees Celsius, interrupted by brief euthermic phases. This study investigates gene expression within the hypothalamus, the key brain area controlling energy balance, adding information on differential gene expression potentially relevant to orchestrate torpor. A *de novo* assembled transcriptome of the hypothalamus was generated from garden dormice hibernating under constant darkness without food and water at 5 °C. Samples were collected during early torpor, late torpor, and interbout arousal. During early torpor, 765 genes were differentially expressed as compared with interbout arousal. Twenty‐seven pathways were over‐represented, including pathways related to hemostasis, extracellular matrix organization, and signaling of small molecules. Only 82 genes were found to be differentially expressed between early and late torpor, and no pathways were over‐represented. During late torpor, 924 genes were differentially expressed relative to interbout arousal. Despite the high number of differentially expressed genes, only 10 pathways were over‐represented. Of these, eight were also observed to be over‐represented when comparing early torpor and interbout arousal. Our results are largely consistent with previous findings in other heterotherms. The addition of a transcriptome of a novel species may help to identify species‐specific and overarching torpor mechanisms through future species comparisons.

## Graphical abstract

Here, we provide hypothalamic transcriptome data for the garden dormouse (*Eliomys quercinus*) at different stages of the torpor arousal cycle (early torpor, late torpor, and interbout arousal) during hibernation season. A *de novo* transcriptome assembly was mapped against *Mus musculus*. Pathways involved in hemostasis, extracellular matrix organization, and signaling of small molecules were overrepresented during torpor.

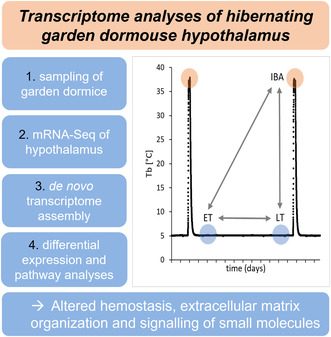

**Abbreviations**
DEG: differentially expressed genes
ET: early torpor
IBA: interbout arousal
log_2_(FC): logarithm of fold change to the base 2
LT: late torpor
*P*adj: adjusted *P*‐value
*T*_b_
: core body temperature

Torpor is the overarching term for metabolic downstate that can be used by endotherms to overcome energetic bottlenecks during harsh environmental conditions [1, 2, 3]. Different forms of torpor exist, ranging from obligate deep hibernation with drastic reduction of metabolic rate, down to 5% of basal, and core body temperature (*T*_b_), reaching single‐digit values for several days or weeks, to more flexible strategies such as spontaneous daily torpor with less severe reductions of metabolic rate and *T*_b_ for only a few hours [1]. Heterotherms adapt physiology, morphology, and behavior, such as body fat mass, development of a more insulating winter fur, nest building, and reproduction prior to the torpor season [4, 5, 6, 7, 8, 9, 10].

During torpor, metabolism and hence all vital functions, such as breathing and heart rate, are drastically reduced [11, 12]. As a consequence, *T*_b_ can drop close to ambient temperature, while some species even hibernate with *T*_b_ values below zero [2, 13]. In rhythmic intervals of days and weeks, hibernating rodents express a so‐called interbout arousal, during which eumetabolism and euthermic values around 37 °C are reached and maintained for a few hours, until the next multiday torpor bout is initiated [14, 15]. Most hibernators such as marmots or dormice exclusively survive the hibernation period on fat reserves, hence overcome a period of long‐term fasting [4], whereas others, such as European hamsters (*Cricetus cricetus*), store food in their hibernaculum [16].

The garden dormouse (*Eliomys quercinus*, Gliridae, Linnaeus 1766) of the European woodlands is a seasonal hibernator that can use torpor when ambient temperature and food availability are low [17, 18]. In preparation for winter, the animals increase their fat stores, remodel their membranes notably in terms of fatty acid composition, group themselves to save energy, and prepare their hibernacula for entering prolonged hibernating bouts during the winter [4, 19, 20]. In autumn, they retreat to their hibernacula until the next spring. During the hibernation period, they show torpor episodes of up to 14 days with *T*_b_ close to ambient temperature that are interrupted by brief interbout arousals of eumetabolism which is maintained and reversed within few hours [16, 21]. The hibernation period can expand up to 6 months, the frequency of interbout arousals is highest in early and late winter, hence, longest torpor bouts are expressed in mid‐winter [17, 22]. In an interplay with this circannual variation with seasons, the ambient temperature fluctuations, age, size, food availability as well as dietary lipid composition influence hibernating patterns [17, 19, 21, 22, 23, 24]. Torpor behavior of the garden dormouse is maintained when the animals are transferred to the laboratory in fall and kept at constant darkness without food and water at 4 °C ambient temperature. At the beginning of a torpor episode, metabolic rate is decreased. In consequence, *T*_b_ drops until it reaches its lowest values of 5 °C. The relationship between oxygen consumption and *T*_b_ has been described in detail [14], so that *T*_b_ can be used to determine metabolic state [25].

The hypothalamus, a central area within the brain, is key in orchestrating metabolic and physiological changes occurring during torpor. This brain part of the diencephalon controls homeostasis of vital functions such as food intake and metabolism, *T*_b_, blood pressure and reproduction during both, in the short‐term as well as in the long‐term hence, over seasons [26]. There is evidence that both, systemic prerequisites to express a metabolic downstate as well as orchestration of metabolic downstate itself are primarily controlled by the hypothalamus [27].

Gene expression profiling studies including the hypothalamus have been done to unravel mechanisms of torpor prerequisites and acute torpor control in different species, including the Djungarian hamster (*Phodopus sungorus*), the European hamster (*C. cricetus*), the 13 lined ground squirrel (*Ictidomys tridecemlineatus*), the golden‐mantled ground squirrel (*Spermophilus lateralis*), and the South American marsupial (*Dromiciops gliroides*) [28, 29, 30, 31, 32, 33, 34]. However, high‐throughput sequencing of the hypothalamus to unravel regulatory mechanisms of metabolic downstates has so far only been done in two species, one obligate hibernator (*I. tridecemlineatus*) and one daily heterotherm (*P. sungorus*) [28, 29, 31]. It is unclear whether different torpor forms are based on similar, species overarching mechanisms and are regulated by similar genes [3, 35, 36, 37]. Next‐generation sequencing data have the potential to further clarify this question once a sufficient number of transcriptome data are available from different species.

In this first comparative transcriptomic study in the garden dormouse, we aimed to characterize differential gene expression within the hypothalamus driven by metabolic state and time spent in torpor. We provide transcriptome data of a novel species to the available data pool, contributing to the puzzle of processes involved in torpor control and providing a valuable source for future comparative approaches, that will be necessary to unravel universal from species specific torpor mechanisms.

## Materials and methods

### Breeding and housing

The garden dormice were issued in 2013 from the outdoors breeding colony at the Research Institute of Wildlife Ecology (University of Veterinary Medicine, Vienna, Austria; latitude 48°15′N, longitude 16°22′E). Offspring were kept in same‐sex groups in large outdoor enclosures with natural ambient temperature and natural photoperiod. Dormice of this experiment were single‐housed indoors at the age of 1.5–2.5 years, during fall at 20 °C ambient temperature in natural photoperiod with food and water *ad libitum* in 60 × 40 × 40 cm cages equipped with bedding and nesting material and branches with leaves, and during winter at 5 °C Ta in constant darkness without food and water in 36 × 20 × 14 cm cages with a customized nest.

### Ethics statement

All procedures regarding dormice experiments were approved by the Ethics and Animal Welfare Committee of the University of Veterinary Medicine, Vienna, in accordance with the University's guidelines for Good Scientific Practice and authorized by the Austrian Federal Ministry of Education, Science and Research (ref BMWF – 68.205/0137‐WF/V/3b/2014) in accordance with current legislation.

### Radiotelemetry

The current metabolic state of each dormouse was assessed with the *T*_b_ provided in real‐time by a radiotelemetry system (DSI – Data Sciences International, Harvard Bioscience Inc., St. Paul, MN, USA). As software, dataquest™ labpro (Data Sciences International, Harvard Bioscience Inc.) was used to monitor *T*_b_ in a resolution of 5 min. A receiver board (RPC‐1) was positioned under every individual home cage. The transmitter model was TA‐10TA‐F10, with a volume of 1.1 cc, a weight of 1.6 g and an accuracy of 0.15 °C, silicone‐coated and manually calibrated from 0 to 40 °C in a water bath. A transmitter was implanted intraperitoneally under anesthesia and analgesia. Anesthesia was induced by subcutaneous injection of 50 mg·kg^−1^ ketamine (Ketamidor 10%; Richter Pharma, Wels, Austria) and 5 mg·kg^−1^ xylazine (Rompun 2%; Bayer, Leverkusen, Germany), and maintained with 1.5% isoflurane via an oxygen stream through a facemask. The implantation of a transmitter into the peritoneal cavity was performed routinely in garden dormice [17, 19, 38] as previously described [39]. For postoperative analgesia, 5 mg·kg^−1^ ketoprofen (Romefen 10% Merial S.A.S., Toulouse, France) was administered subcutaneously. The dormice were implanted during the pre‐hibernation phase.

### Sampling scheme

**Fig. 1:**
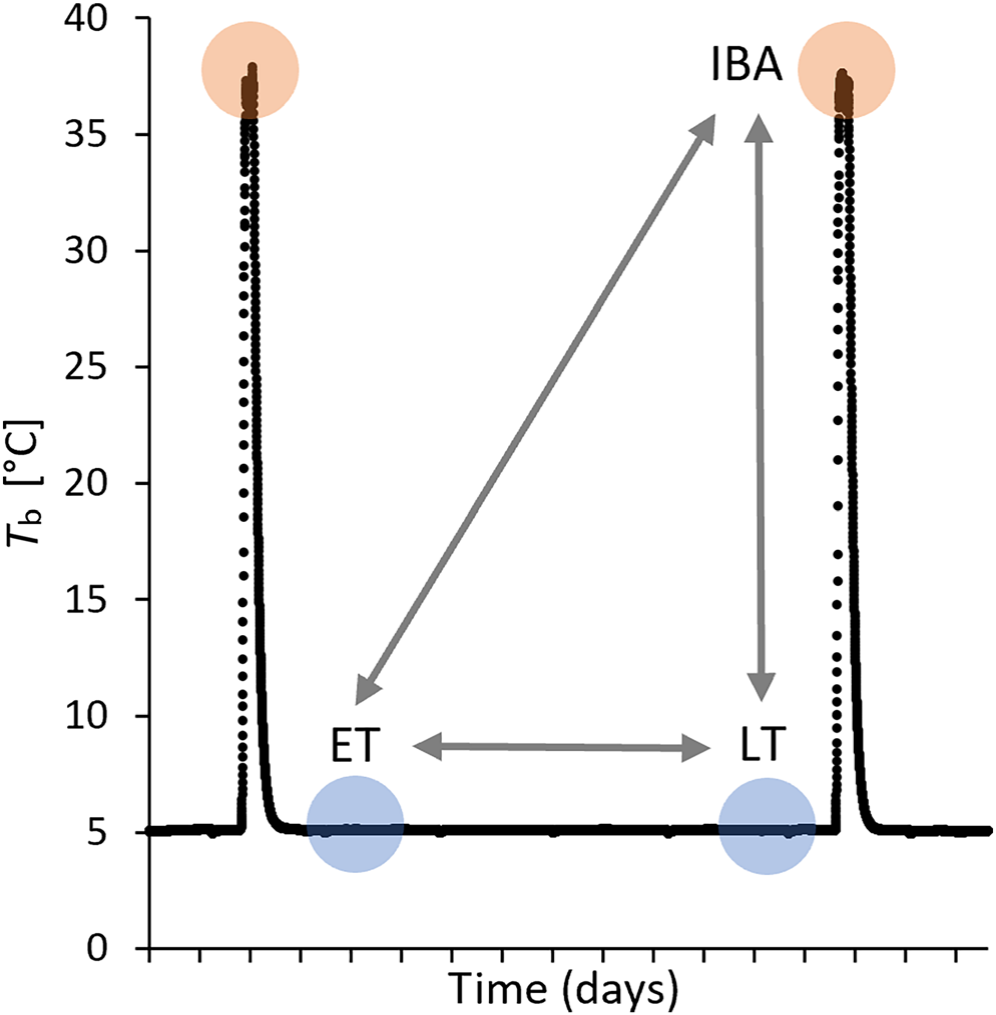
Sampling scheme. Core body temperature (*T*_b_) of one dormouse over the course of 2 weeks during hibernation in constant darkness without food or water at 5 °C ambient temperature. Animals were sacrificed in early torpor (ET, 1 or 2 days in torpor, *n* = 4), late torpor (LT, 9 or 10 days in torpor, *n* = 3), or interbout arousal (IBA, 2 of 4 h in interbout arousal, *n* = 4). Radiotelemetry data were used previously [19, 39]. Transcriptome data of sampling groups were compared pairwise (arrows; ET vs LT, ET vs IBA, LT vs IBA).

Hibernation was induced by removing food and water and housing animals at 5 °C in cooling units. Sacrifices occurred after several weeks of hibernation in mid‐winter when torpor bout duration is maximal. The points of sacrifice are shown in Fig. 1. Twelve dormice (six females, six males; 89.8 ± 10.8 g body mass) were sacrificed in 2015 in their second or third winter by immediate decapitation in early torpor (ET, *T*_b_ = 5 °C for 36 h, *n* = 4, one female, three males), late torpor (LT, *T*_b_ = 5 °C for 230 h, *n* = 4, two females, two males) and, after loss of consciousness when exposed to carbon dioxide followed by immediate decapitation, in interbout arousal (IBA, *T*_b_ = 37 °C for 4 h, *n* = 4, three females, one male). Implanted transmitters revealed a *T*_b_ of 4.9 ± 0.5 °C in torpid (*n* = 7) and 36.8 ± 0.4 °C in euthermic animals (*n* = 4) at an ambient temperature of 5 °C. Bioinformatic scripts are openly available in the Open Access Repositorium of Ulm University OPARU [40]. Table S1 contains background information for each animal. The animals of this study were included in a large project with the aim to characterize adaptations of the immune system during hibernation [39]. Immediately after decapitation of the animals, brains were removed carefully and directly flash‐frozen and then stored at −80 °C until further analyses.

### Lab work

Hypothalamic blocks were cut from frozen brains at −20 °C. Brains were placed on the dorsal side and the hypothalamus was dissected between the optic chiasm as rostral, mammillary bodies as caudal and hypothalamic sulci as lateral borders. The anterior commissure was used as dorsal border. Hypothalamic blocks were stored again at −80, before being processed together for sequencing. Samples were homogenized in 3 mL TriFast by using a micropestle. Total RNA was isolated using peqGOLD Trifast™ (Peqlab, Erlangen, Germany) and purified using the Crystal RNA MiniKit (Biolabproducts, Bebensee, Germany) including an on‐column digestion with RNase‐free DNase (Qiagen, Hilden, Germany). RNA purity was assessed by the 260/280 nm ratio on a NanoDrop 1000 spectrophotometer. RNA integrity was proven by formaldehyde agarose gel electrophoresis. The quantity was between 1.0 and 1.4 μg (1.2 ± 0.1 μg) and the concentration was between 210 and 667 ng·μL^−1^ (446 ± 143 ng·μL^−1^). The RIN varied between 6.2 and 7.3 (6.6 ± 0.4). mRNA‐Seq was conducted in 2017 on an Illumina NextSeq 500 (paired end, 4 lanes) by StarSEQ GmbH, Mainz, Germany. The raw Illumina data that support the findings of this study are openly available in NCBI's Sequence Read Archive SRA and are accessible through https://www.ncbi.nlm.nih.gov/geo/query/acc.cgi?acc=GSE207494, GEO Series accession number GSE207494 [41].

### Data processing

The bioinformatics pipeline used in this study is based on the pipeline developed in Haugg *et al*. [29]. Bioinformatic analyses were performed on the bwForCluster NEMO of the Baden‐Württemberg High Performance Computing (bwHPC) project. The quality of raw RNA‐Seq data was evaluated using fastqc 0.11.9f (https://www.bioinformatics.babraham.ac.uk/projects/fastqc/) and no QC issues were detected. All reads have a sequence length of 151 base pairs. The GC content was 48–49% for all sequencing runs. The number of read pairs per sample ranged from 26 Mio. to 38 Mio. with a mean of 33 Mio. Table S2 contains the number of read pairs per sample [40]. trim‐galore 0.6.6 (https://github.com/FelixKrueger/TrimGalore) was used to trim remaining adapter sequences from the raw data. A *de novo* transcriptome assembly was prepared using trinity 2.8.5 [42, 43] using data from all 12 dormice (395 143 505 read pairs in total). blastx 2.5.0+ (https://www.ncbi.nlm.nih.gov/books/NBK279688/) with an *e*‐value cut‐off < 1E‐5 was used to map the transcripts against the reference proteome of *Mus musculus* (GRCm39, Annotation Release 109) [44, 45, 46]. Using “extract_hits_from_fasta.rb”, a reduced assembly containing only transcripts with a hit against a mouse protein was generated. Using “hits_to_genemap.rb”, a transcript‐to‐GeneID dictionary was generated based on the best hit to the mouse proteome. Both ruby‐scripts are publicly available in SourceForge at https://sourceforge.net/projects/prepare‐transcript‐to‐gene‐map/. bowtie 1.3.0 was used to map back the reads from each sample to the reduced assembly [47]. rsem 1.3.1 was used to calculate non‐normalized differential gene expression per sample [48]. Both the original and the processed *de novo* assembly as well as the non‐normalized gene expression tables are openly available in NCBI's Gene Expression Omnibus and are accessible through https://www.ncbi.nlm.nih.gov/geo/query/acc.cgi?acc=GSE207494, GEO Series accession number GSE207494 [41]. The bioinformatics pipeline from raw Illumina data to non‐normalized results is openly available as bash commands in (OPARU) [40].

### Identification of hypothalamus‐specific genes

To verify that the dissections did indeed yield hypothalamic tissue, normalized mouse expression data were downloaded from the Human Protein Atlas (https://www.proteinatlas.org/about/download) for 13 brain regions. From this data, nine hypothalamus‐specific genes that have an expression > 10 TPM in the mouse hypothalamus and at least four‐fold higher mRNA levels in hypothalamus compared to all other regions were selected. The expression levels of these genes were then compared between the mouse hypothalamus and the dormouse samples. Scripts are available in (OPARU) [40].

### Differential gene expression analysis

The calculated transcript expression values were aggregated at gene‐level using the mouse GeneIDs that were assigned using blastx. Each GeneID includes all isoforms, precursors, and preproproteins of that gene. Only genes with a mean total read count of at least 10 in at least one of the three experimental groups were reported. The pairwise comparison of the normalized counts per group resulted in the gene's fold change, provided as log_2_(FC), and the significance of this fold change (adjusted *P*‐value, *P*adj). Genes with a *P*adj < 0.05 were defined as differentially expressed (DEGs). Fold changes are given for the first named group relative to the second named group. One animal (LT01, female, terminal *T*_b_: 4.09 °C) was excluded from the analyses because it had an undetectable RIN (Table S3) [40].

Statistical analyses including the principal component analyses and the hierarchical clustering were performed with deseq2 [49]. Data were normalized across all samples. In this study, three pairwise group comparisons were conducted: ET (*n* = 4) vs IBA (*n* = 4), ET (*n* = 4) vs LT (*n* = 3), and LT (*n* = 3) vs IBA (*n* = 4). Sex was included as a covariate in the statistical model for all analyses of differential expression. The statistical pipeline comprising normalization of data across all samples, pairwise group comparison, principal component analysis, and heatmap with hierarchical clustering is available in (OPARU) [40]. Data were processed using Microsoft Excel. Figures were generated with Microsoft Excel (Office 365, 2016), except Fig. 3, hierarchical clustering, which was generated using rstudio 3.5.2 (https://www.r‐project.org/, http://www.rstudio.com/).

To identify overrepresented reactome pathways (Reactome version 77, released 2021‐10‐01), GO overrepresentation analyses (PANTHER Overrepresentation Test, released 2022‐10‐13) were performed at http://pantherdb.org/tools/compareToRefList.jsp with Fisher's Exact as test type and False Discovery Rate (FDR) correction [50, 51, 52]. For each pairwise group comparison, DEGs that were increased (*P*adj < 0.05 and log_2_(FC) > 1) and decreased (*P*adj < 0.05 and log_2_(FC) < 1) were tested separately. This approach has previously been shown to increase the statistical power as compared to using all DEGs in the same enrichment analyses due to imbalances between increased and decreased DEGs in particular pathways [53]. All present genes (any *P*adj‐value, any log_2_(FC)‐value) served as background. Hierarchical ranking of overrepresented pathways was based on https://reactome.org/ [54].

The data were screened for genes involved in hypothalamic systems with potential roles in torpor control. A list of 68 previously defined genes referred to as “indicator genes” was used [29].

## Results

### *De novo* assembly of transcriptomes and mapping

On average, 32 928 625 read pairs per sample were sequenced. In total, 395 143 505 read pairs from all samples were pooled to generate a single *de novo* transcriptome assembly of the garden dormouse hypothalamus comprising 1 382 822 transcripts. Values per sample are listed in Table S2.

Using a blastx search against the mouse proteome, 16 798 distinct protein coding genes were identified in the assembly corresponding to 73% of the entire gene repertoire of the mouse (22 177 protein‐coding genes). The mean bit‐score was 484.3 with 90% of all transcripts falling in the range of 49.3–1678. In total, 164 435 transcripts (median length 1515 bp) were successfully annotated using this approach (the median length of transcripts without a significant blast hit was 374 bp) (Fig. S1, scripts (OPARU)). Nine genes were identified to be highly specific for the hypothalamus in mice. The expression of these genes is similar or significantly higher in all dormouse samples compared to the mouse hypothalamus confirming the specificity of the dissection (Fig. S2, scripts (OPARU)).

### Differential gene expression

**Fig. 2:**
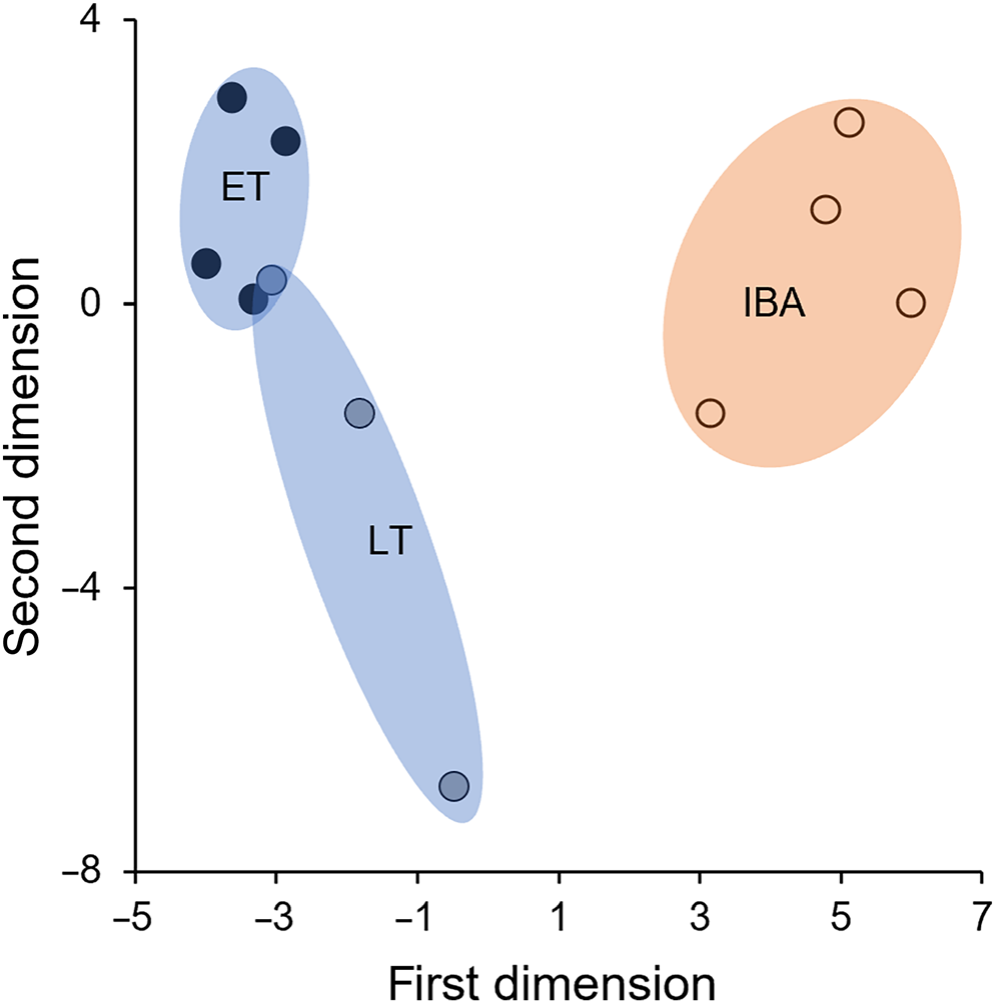
Principal component analysis. Distance between samples of this study (dots; ET: black, LT: blue, IBA: orange) by their respective overall gene expression in the first dimensions (PC1: 27% variance, PC2: 13% variance). Data basis is ET (*n* = 4), LT (*n* = 3), and IBA (*n* = 4).

The principal component analysis shows distinct clustering of the experimental groups. Samples taken during early torpor clustered in a tight area. Samples taken during late torpor clustered nearby, spanning a wider value range in the second dimension. The cluster of samples taken during interbout arousal substantially differed in the first dimension from samples gained in both early and late torpor, but not in the second dimension (Fig. 2).

**Fig. 3:**
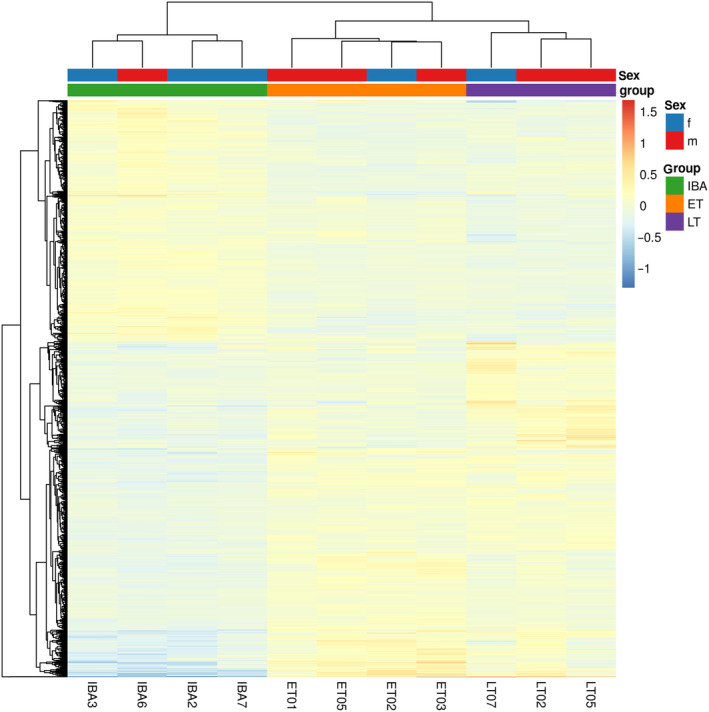
Heatmap and hierarchical clustering of genes differentially expressed in at least one pairwise group comparison. Gene expression (gradient from blue, low, to red, high expression) of 1351 DEGs (*y*‐axis) with *P*adj < 0.05 in at least one pairwise group comparison is plotted for each of the 11 samples (*x*‐axis). Genes are listed in File S1.

**Fig. 4:**
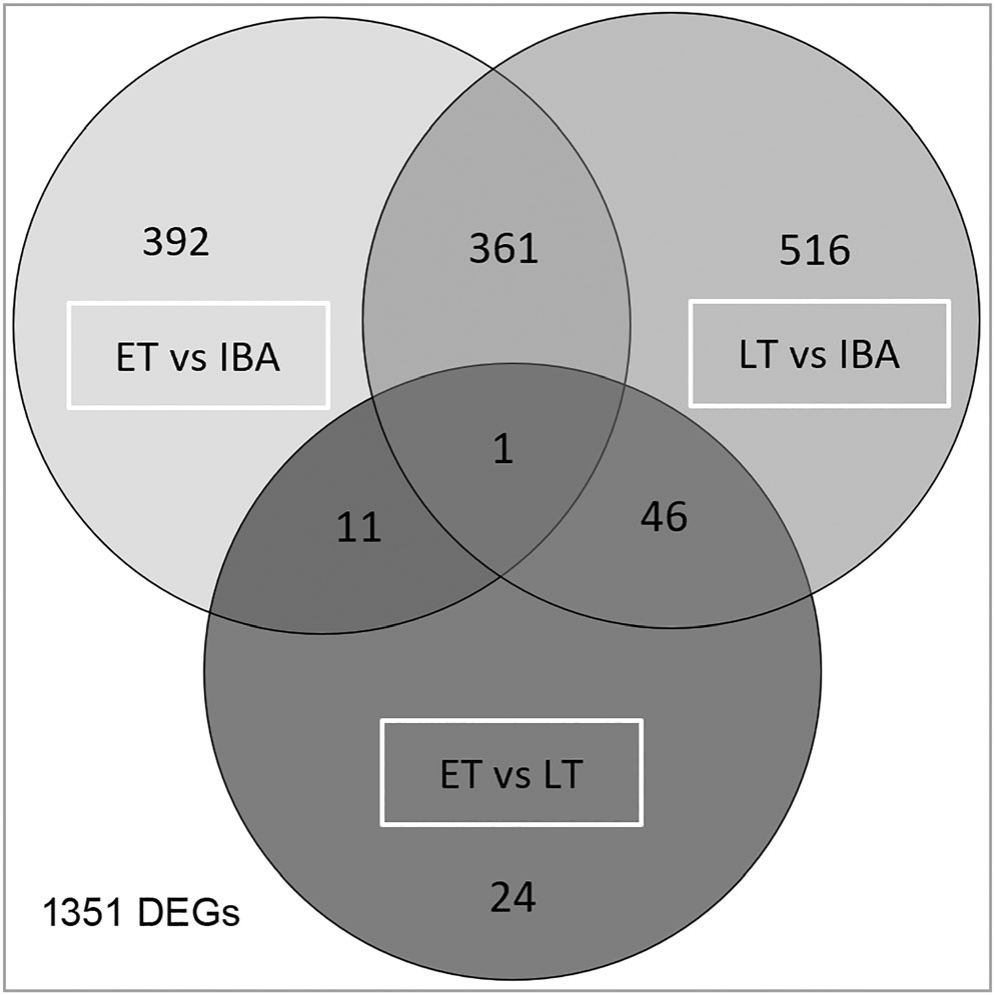
State‐specific genes. Number of DEGs with *P*adj < 0.05 and any fold change shared between or specific for the pairwise group comparisons ET vs IBA, ET vs LT, and LT vs IBA and their intersections. Genes per intersection are listed in File S1.

A hierarchical clustering for all 1351 genes that were differentially expressed in at least one pairwise group comparison recovered the three sampling groups early torpor (ET), late torpor (LT), and interbout arousal (IBA) as distinct clusters irrespective of sex (Fig. 3). While 392, 24 and 516 genes were exclusively differentially expressed in ET vs IBA, ET vs LT, and LT vs IBA, respectively, 419 genes showed differential expression in more than one pairwise group comparison (Fig. 4, File S1).

**Fig. 5:**
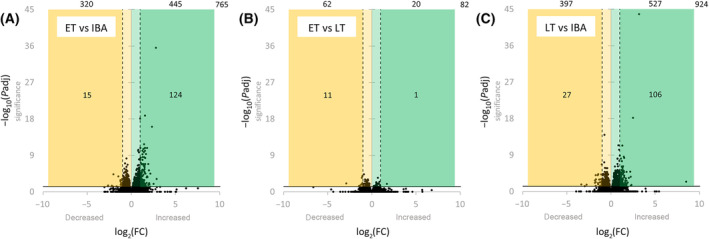
Volcano plots. Differential gene expression for the pairwise group comparisons ET vs IBA (A), ET vs LT (B), and LT vs IBA (C). All annotated genes (dots) are plotted based on fold change (*x*‐axis) and significance (*y*‐axis). DEGs (*P*adj < 0.05) are shown above the horizontal solid line (−log_10_(0.05) = 1.3), while DEGs with a high significance (*P*adj < 0.001) are shown above the dotted line (−log_10_(0.001) = 3.0). Genes with decreased expression log_2_(FC) < 0 are shown within the yellow area, while those with a decreased expression of log_2_(FC) < −1 are shown within the dark yellow area. Genes with increased expression log_2_(FC) > 0 are shown within the green area, while those with increased expression of log_2_(FC) > 1 are shown within the dark green area. The numbers of DEGs per area are indicated, while the number in the right upper corner states the total number of DEGs. Data basis is ET (*n* = 4), LT (*n* = 3), and IBA (*n* = 4). Genes are listed in File S1.

In ET compared to IBA, 5.1% of all present genes were differentially expressed. Of these 765 DEGs, expression of 445 genes was increased, and of 320 genes decreased. This accounts for a proportion of 6 : 4 of increased to decreased DEGs. 139 (18.1%) of the 765 DEGs had a log_2_(FC) > 1. Comparing ET to LT, 82 genes (0.6%) were differentially expressed (20 increased and 62 decreased, proportion 2 : 8). Of these 82 genes, 12 (14.6%) had a log_2_(FC) > 1. In LT compared to IBA, 924 genes (6.2%) were differentially expressed (527 increased and 397 decreased, proportion 6 : 4) (Fig. 5, File S1). Of the 924 genes, 133 (14.4%) had a |log_2_(FC)| > 1. ET vs IBA and LT vs IBA had 362 DEGs in common, which had the same direction of change; expression of 202 genes increased, and expression of 160 genes decreased in both pairwise group comparisons (Fig. 4, File S1).

### Pathway analysis

Across the three pairwise group comparisons, 29 reactome pathways were enriched by the overexpressed genes. In ET vs IBA, 27 pathways were overrepresented, none in ET vs LT, and 10 in LT vs IBA. In ET vs IBA, the overrepresented pathways belonged to the high‐ranking pathways hemostasis (4) and extracellular matrix organization (10). Moreover, pathways associated with signal transduction (4), transport of small molecules (7), metabolism of proteins (1), and programmed cell death (1) were overrepresented (Table 1). In LT vs IBA, 10 pathways were overrepresented, eight of which were also enriched in ET vs IBA. These shared pathways were associated with hemostasis (2 of 4), extracellular matrix organization (4), and transport of small molecules (2). Two pathways were exclusively enriched in LT vs IBA, namely response to elevated platelet cytosolic Ca^2+^ and platelet degranulation associated with hemostasis (Table 2). The comprehensive tables of enriched pathways are provided in File S2.

**Table 1:** Overrepresented reactome pathways in ET vs IBA. Pathways overrepresented by the 124 increased differentially expressed genes with *P*adj < 0.05 and log_2_(FC) > 1. All 14 862 mapped genes served as background. Pathways were sorted in blocks according to each hierarchically highest pathway (bold) with its sub‐pathways (each hierarchical level indicated with “‐”). Pathways with false discovery rate (FDR) < 0.05 were taken as significant (color code from white, low value, to red, high value). Pathways indicated as not significant (n.s., gray) had an FDR > 0.05 and serve as orientation. Comprehensive results are given in File S2.

FDR	Reactome pathway	
n.s.	R‐MMU‐109582	**Hemostasis**
4.65E‐02	R‐MMU‐76002	‐ Platelet activation, signaling, and aggregation
1.94E‐03	R‐MMU‐76009	‐ ‐ Platelet Aggregation (Plug Formation)
1.44E‐02	R‐MMU‐430116	‐ ‐ GP1b‐IX‐V activation signaling
1.00E‐02	R‐MMU‐75892	‐ Platelet Adhesion to exposed collagen
4.95E‐07	R‐MMU‐1474244	**Extracellular matrix organization**
6.01E‐08	R‐MMU‐3000178	‐ ECM proteoglycans
3.06E‐02	R‐MMU‐3000171	‐ Non‐integrin membrane‐ECM interactions
1.38E‐05	R‐MMU‐216083	‐ Integrin cell surface interactions
1.03E‐02	R‐MMU‐1474290	‐ Collagen formation
3.69E‐03	R‐MMU‐1650814	‐ ‐ Collagen biosynthesis and modifying enzymes
5.76E‐04	R‐MMU‐8948216	‐ ‐ ‐ Collagen chain trimerization
1.80E‐03	R‐MMU‐2022090	‐ ‐ Assembly of collagen fibrils and other multimeric structures
7.16E‐04	R‐MMU‐1474228	‐ Degradation of the extracellular matrix
5.91E‐04	R‐MMU‐1442490	‐ ‐ Collagen degradation
n.s.	R‐MMU‐162582	**Signal transduction**
4.43E‐02	R‐MMU‐9006934	‐ Signaling by Receptor Tyrosine Kinases
3.97E‐02	R‐MMU‐6806834	‐ ‐ Signaling by MET
4.15E‐03	R‐MMU‐8875878	‐ ‐ ‐ MET promotes cell motility
1.60E‐03	R‐MMU‐8874081	‐ ‐ ‐ ‐ MET activates PTK2 signaling
3.52E‐02	R‐MMU‐382551	**Transport of small molecules**
5.60E‐04	R‐MMU‐425407	‐ SLC‐mediated transmembrane transport
5.09E‐04	R‐MMU‐425366	‐ ‐ Transport of bile salts and organic acids, metal ions and amine compounds
4.60E‐02	R‐MMU‐561048	‐ ‐ ‐ Organic anion transport
2.51E‐02	R‐MMU‐442660	‐ ‐ ‐ Na+/Cl− dependent neurotransmitter transporters
1.40E‐02	R‐MMU‐425393	‐ ‐ Transport of inorganic cations/anions and amino acids/oligopeptides
1.61E‐02	R‐MMU‐352230	‐ ‐ ‐ Amino acid transport across the plasma membrane
n.s.	R‐MMU‐392499	**Metabolism of proteins**
6.19E‐04	R‐MMU‐381426	‐ Regulation of Insulin‐like Growth Factor (IGF) transport and uptake by Insulin‐like Growth Factor Binding Proteins (IGFBPs)
n.s.	R‐MMU‐5357801	**Programmed cell death**
1.07E‐02	R‐MMU‐351906	‐ Apoptotic cleavage of cell adhesion proteins

**Table 2:** Overrepresented reactome pathways in LT vs IBA. Pathways overrepresented by the 106 increased differentially expressed genes with *P*adj < 0.05 and log_2_(FC) > 1. All 14 862 mapped genes served as background. Pathways were sorted in blocks according to each hierarchically highest pathway (bold) with its sub‐pathways (each hierarchical level indicated with “‐”). Pathways with false discovery rate (FDR) < 0.05 were taken as significant (color code from white, low value, to red, high value). Pathways indicated as not significant (n.s., gray) had an FDR > 0.05 and serve as orientation. Comprehensive results are given in File S2.

FDR	Reactome pathway	
n.s.	R‐MMU‐109582	**Hemostasis**
3.38E‐02	R‐MMU‐76002	‐ Platelet activation, signaling, and aggregation
2.85E‐02	R‐MMU‐76009	‐ ‐ Platelet aggregation (plug formation)
3.00E‐02	R‐MMU‐76005	‐ ‐ Response to elevated platelet cytosolic Ca^2+^
2.67E‐02	R‐MMU‐114608	‐ ‐ ‐ Platelet degranulation
2.83E‐02	R‐MMU‐1474244	**Extracellular matrix organization**
2.73E‐03	R‐MMU‐3000178	‐ ECM proteoglycans
3.37E‐02	R‐MMU‐1474228	‐ Degradation of the extracellular matrix
9.19E‐03	R‐MMU‐216083	‐ Integrin cell surface interactions
n.s.	R‐MMU‐382551	**Transport of small molecules**
7.29E‐03	R‐MMU‐425366	‐ Transport of bile salts and organic acids, metal ions and amine compounds
3.30E‐02	R‐MMU‐442660	‐ ‐ Na+/Cl− dependent neurotransmitter transporters

### Screening for indicator genes

**Fig. 6:**
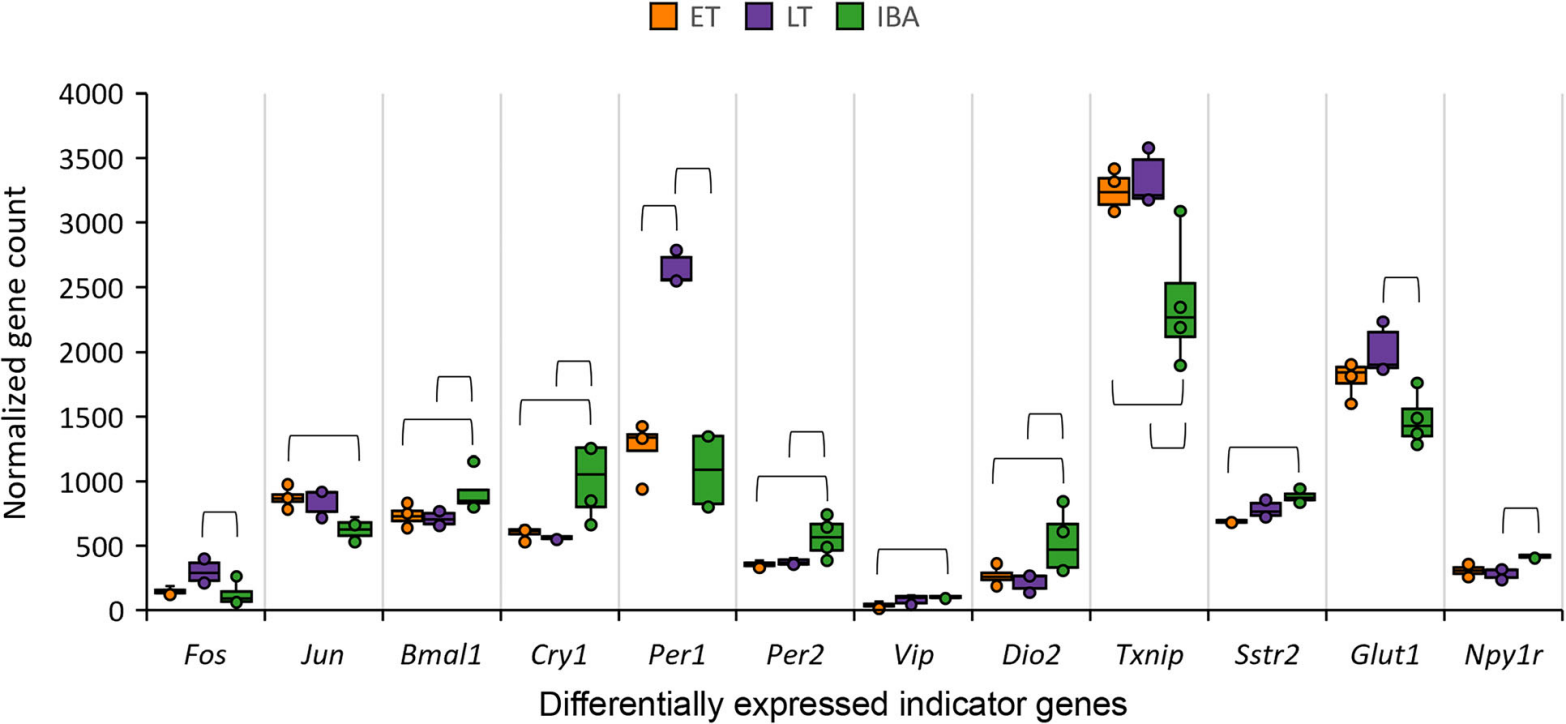
Indicator genes differentially expressed in at least one pairwise group comparison. To visualize differences between samples within a group and differences between groups, the normalized count was plotted for all differentially expressed indicator genes with *P*adj < 0.05 in at least one pairwise group comparison. Data basis is ET (*n* = 4), LT (*n* = 3), and IBA (*n* = 4). Significant differences between groups are indicated with brackets. Associated *P*adj‐values are listed in Table 3. Results of all 68 indicator genes screened for are provided in File S3.

Data were screened for genes involved in hypothalamic systems with potential roles in torpor control [29]. Of these 68 tested, previously defined “indicator genes”, eight were differentially expressed in ET vs IBA (transcription: *Jun*; circadian clock: *Bmal1*, *Cry1*, *Per2*, *Vip*; thyroid system: *Dio2*, *Txnip*; growth axis: *Sstr2*), one gene was differentially expressed in ET vs. LT (circadian clock: *Per1*), and nine were differentially expressed in LT vs IBA (transcription: *Fos*; circadian clock: *Bmal1*, *Cry1*, *Per1*, *Per2*; thyroid system: *Dio2*, *Txnip*; metabolism: *Glut1*, *Npy1r*) (Fig. 6, Table 3, File S3).

**Table 3:** Differentially expressed indicator genes. Data basis is ET (*n* = 4), LT (*n* = 3), and IBA (*n* = 4). Those indicator genes differentially expressed with *P*adj < 0.05 out of a previously defined list with 68 genes [29]. The color code indicates decreased genes with log_2_(FC) < 0 (yellow), increased genes with log_2_(FC) > 0 (green), and genes differentially expressed in two of the three pairwise comparisons ET vs IBA, ET vs LT, and LT vs IBA (red). Normalized counts per indicator gene and sample are plotted in Fig. 6. Results of all 68 indicator genes screened for are provided in File S3.

	Indicator		Differentially gene expression			Gene		
	System	ID	*P*adj	−log_10_(*P*adj)	log_2_(FC)	ID	Symbol	Product
ET vs IBA	Transcription	3	0.028	1.6	0.47	16 476	*Jun*	Transcription factor AP‐1 / c‐Jun
	Clock	10	0.041	1.4	−0.41	11 865	*Bmal1*	Brain and muscle ARNT‐like 1
		14	0.003	2.6	−0.81	12 952	*Cry1*	Cryptochrome‐1
		22	0.014	1.8	−0.71	18 627	*Per2*	Period circadian protein homolog 2
		27	0.000	4.3	−2.01	22 353	*Vip*	Vasoactive intestinal peptide
	Thyroid	29	0.001	2.8	−1.23	13 371	*Dio2*	Iodothyronine deiodinase type II
		38	0.034	1.5	0.45	56 338	*Txnip*	Thioredoxin‐interacting protein
	Growth	42	0.024	1.6	−0.31	20 606	*Sstr2*	Somatostatin receptor type 2
ET vs LT	Clock	21	0.000	3.8	−1.07	18 626	*Per1*	Period circadian protein homolog 1
LT vs IBA	Transcription	1	0.050	1.3	1.29	14 281	*Fos*	Proto‐oncogene c‐Fos
	Clock	10	0.026	1.6	−0.44	11 865	*Bmal1*	Brain and muscle ARNT‐like 1
		14	0.001	3.0	−0.90	12 952	*Cry1*	Cryptochrome‐1
		21	0.000	6.1	1.24	18 626	*Per1*	Period circadian protein homolog 1
		22	0.043	1.4	−0.62	18 627	*Per2*	Period circadian protein homolog 2
	Thyroid	29	0.000	4.0	−1.51	13 371	*Dio2*	Iodothyronine deiodinase type II
		38	0.022	1.7	0.48	56 338	*Txnip*	Thioredoxin‐interacting protein
	Metabolism	48	0.026	1.6	0.38	20 525	*Glut1*	Glucose transporter member 1
		63	0.024	1.6	−0.54	18 166	*Npy1r*	Neuropeptide Y receptor type 1

## Discussion

### *De novo* assembly of transcriptomes and mapping

In this first comparative transcriptomic study in the garden dormouse, we generated a *de novo* assembly of a novel species and found distinct gene expression changes at different stages of a torpor arousal cycle.

All hypothalamic samples taken during early torpor, late torpor, or interbout arousal during the hibernation season contributed to the *de novo* assembly with 395 143 505 read pairs (Fig. 1, Table S2). Using the mouse genome as reference, 164 435 garden dormouse transcripts could successfully be annotated. These transcripts were assigned to 16 798 distinct protein coding genes representing 76% of the entire protein coding gene repertoire of the mouse (File S1). The transcripts that could not be annotated this way were significantly shorter than the annotated transcripts (median length 1515 bp vs 374 bp) and the majority likely represent short assembly fragments that did not contain sufficient sequence information for a confident match to a different species (Fig. S1). The longer unannotated transcripts may either come from genes not present in the mouse or from fast evolving genes that have diverged to a point where sequence similarity at the amino acid level is insufficient for identification using blastx [55]. Although our approach of annotating transcripts using assignment to the proteome of a different species is bound to lose a set of species‐specific transcripts and non‐protein coding genes such as lncRNAs, miRNAs or snoRNAs, the data provide a first impression of gene expression changes in the hibernating garden dormouse. The complete *de novo* assembly of a novel species will be a valuable resource for the community and future, comparative analyses between species [41].

### Differential gene expression

Samples clustered according to state in the principal component analysis and the heatmap with hierarchical clustering analyses (Figs 2 and 3). The IBA samples formed a distinct cluster indicating a clear separation in expression patterns between torpor and interbout arousal, which has also previously been described in different brain areas of the 13‐lined ground squirrel (*I. tridecemlineatus*) [31]. ET and LT clustered closely together, with LT showing highest variability in the second dimension. This might reflect the less precise state definition for LT, since samples were collected at a defined time point relative to torpor entry without knowing the exact time of the next arousal phase which may vary between animals (Fig. 1, Table S1). Shifts in the expression profile, however, might be linked to the time of the next arousal.

Our data set comprises samples of animals during the hibernation seasons at different stages of a torpor arousal cycle only, without summer animals being available. We are aware, that many hypothalamic systems are being remodeled in a seasonal context, which is likely to be a prerequisite for torpor to occur at all [55, 56]. Nevertheless, the transcriptional changes between different stages across the torpor arousal cycle will reflect the acute regulatory and/or adaptive changes in the hypothalamus, that have been even less understood.

As expected, gene expression changes in the garden dormouse’ hypothalamus were most pronounced between both torpor states and IBA, and less pronounced within torpor (ET vs LT). Of 16 798 annotated genes, 765 (5.1%), 82 (0.6%), and 924 (6.2%) genes were differentially expressed in ET vs IBA, ET vs LT, and LT vs IBA, respectively. The proportion of increased to decreased DEGs was 6 : 4 comparing torpor with IBA (both ET vs IBA and LT vs IBA) but 3 : 7 within torpor (ET vs LT). In the hypothalamus of the 13‐lined ground squirrel (*I. tridecemlineatus*), the proportion of increased to decreased DEGs was 6 : 4 in torpor entry and 7 : 3 in torpor arousal, each compared with IBA, but 2 : 8 in the within‐torpor comparison (torpor entry vs late torpor) [31]. Similar ratios of increased and decreased DEGs have been found in different heterotherms [28, 29, 31, 34, 57]. Transcriptional and translational processes are highly temperature sensitive [57, 58, 59, 60]. High numbers of genes with increased expression will mostly result from decreased translation and therefore accumulation of mRNA and will strongly be influenced by stabilization processes [31, 61]. This complicates interpretation of next generation sequencing data alone. However, there is evidence, that the hypothalamus remains more active than other brain regions and that at least distinct hypothalamic nuclei persist the low *T*_b_s and maintain some transcriptional activity [31, 62, 63].

### Pathway analysis

In ET vs IBA, 27 pathways were overrepresented by the 445 genes with increased expression (Table 1, File S2). Given the transcriptional‐translational temperature sensitivity it remains unclear whether transcripts are translated into proteins during torpor [57, 58, 59]. Nevertheless, overrepresentation of distinct transcriptional pathways may reflect biologically relevant remodeling of the given process, providing useful starting points for further investigations at protein level.

A possible example for accumulation of mRNA might be the hemostasis sub‐pathways that were overrepresented in ET vs IBA. Hemostasis is the physiological process that stops bleeding after vessel injury, including platelet activation, aggregation, adhesion to the injured vessel wall, and thrombus formation [64, 65, 66]. Hibernators, however, are able to withstand periods of low blood flow during torpor and quick and vigorous reperfusion during arousal without thrombi formation and ischemia [67, 68, 69]. Changes in platelet count as well as selective decrease in gene expression and activity of different blood clotting factors were shown in the lung during torpor, all contributing to decreased thrombosis risk [70, 71]. Our animals were not perfused with saline prior to sampling, hence included blood cells within the tissue. Consequently, it could be speculated, that enrichment of mRNA related to clot formation either results from attenuated translational events to prevent ischemic events during torpor, or from altered ratio of blood to brain tissue resulting from the low blood flow and viscous blood during torpor.

Genes involved in the extracellular matrix organization pathway have previously been described to be differentially expressed during torpor in the hypothalamus of heterotherms, including integrin signaling as well as collagen modifications. In the brain, the extracellular matrix constitutes 10–20% of brain volume and plays major roles in maintenance of the blood–brain barrier, structuring of cells into distinct regions by providing anchorage to cells, axonal guiding, and regulating synaptic plasticity [72]. Integrins are membrane proteins linking the cytoskeleton to the extracellular matrix. They play an important role in migration, proliferation, and cell survival, hence neuroplasticity. An increase of integrin coding gene transcripts has previously been found in the hibernating 13‐lined ground squirrels (*I. tridecemlineatus*) and has been discussed to facilitate structural changes in the brain and their fast initialization and reversal during arousal [32, 73, 74, 75]. This is further supported by the signaling by receptor kinases pathway, specifically MET activated PTK2 (FAK1) signaling, that has closely been associated with integrin‐mediated cell motility [76, 77, 78, 79]. Collagens are involved in brain architecture, including axonal guidance and synaptogenesis and differential expression of collagen‐related genes has previously been described in brain transcriptomes of Djungarian hamsters (*P. sungorus*) and 13‐lined ground squirrels (*I. tridecemlineatus*) [28, 31, 32]. Overrepresentation of both, pathways involved in collagen formation as well as collagen degradation, suggests a precisely balanced collagen reorganization during torpor. Taken together, tightly controlled extracellular matrix reorganization combined with distinct MET signaling may likely contribute to enhanced neuroprotection and neuroplasticity during torpor.

Furthermore, transport of small molecules with subpathways were overrepresented in ET vs IBA, including solute carrier (SLC) transmembrane transport. SLCs are the largest family of transmembrane transporters and control transport of multiple substances including ions, amino acids and nutrients across membranes. In the brain, they are expressed in the endothelial cells of the blood–brain barrier, choroid plexus as well as in neurons and astrocytes. In the blood–brain barrier, SLCs play roles in protecting the brain from detrimental, but absorbing essential substances from the blood. In neurons and astrocytes, they are involved in regulating neurotransmission and modulation [80]. Overrepresentation of the subpathways amino acid transport, as well as Na+/Cl− dependent neurotransmitter transporters, may support distinct remodeling of both, blood–brain barrier as well as neurotransmission.

Five hundred and twenty‐seven genes with increased expression in LT vs IBA including the 202 genes with increased expression shared between the torpid states (Table 2, File S2), 10 pathways were enriched, eight of which were shared with ET vs IBA and belong to the high‐ranking pathways hemostasis, extracellular matrix organization and transport of small molecules. However, each of the high‐ranking pathways in LT vs IBA was supported by a smaller number of sub‐pathways than in ET vs IBA (Tables 1 and 2, File S2). One possible explanation for this finding, together with the higher variance in the clustering analysis, is the less precise state definition for LT, that was sampled relative to torpor entry without knowing the exact time of the next arousal for each individual. It might also suggest that gene expression of potentially torpor relevant mechanisms, shifts over the duration of the torpor bout and becomes less distinct in LT vs IBA. This might result from temperature‐driven effects on the transcriptional/translational machinery that may accumulate over time, and “disorganize” torpor relevant processes. It has previously been discussed, that transcript of a “hypothetical hibernation gene” might be degraded during torpor, until a threshold is reached that triggers arousal, during which transcription can be replenished [31]. In this line it is tempting to speculate, that less distinct organization of torpor critical pathways might eventually cause arousal.

### Screening for indicator genes

The data set was screened for 68 indicator genes potentially relevant for torpor (Fig. 6, Table 3, File S3) [29]. Five differentially expressed indicator genes were genes of the circadian system, four of which were decreased during torpor. The latter is consistent with previous gene expression studies of the circadian system in the Artic ground squirrel (*Urocitellus parryii*) and the European hamster (*C. cricetus*), demonstrating that clock genes in the suprachiasmatic nucleus, stop cycling during hibernation and regain rhythmicity upon arousal [30, 81, 82]. The only exception in our data was *Per1*, showing low mRNA levels in ET and IBA and high levels in LT, suggesting an oscillation of *Per1* during torpor (Fig. 6). We can only speculate whether this discrepancy might result from species‐specific clock regulation or the potentially very soon upcoming arousal, prior to which *Per1* transcription might be initiated to quickly regain rhythmicity during IBA. Certainly, the limited number of sampling points irrespective of circadian biology and the unknown circadian phase of the IBA sampling point impedes a thorough interpretation.

In our data set, transcripts of the transcription factors *Jun* and *Fos* were increased during torpor. *Jun* and *Fos* act as heterodimers on AP‐1 sites of target genes. Transcription factors like *Jun* and/or *Fos* have previously been shown to be increased in both, whole hypothalamus samples as well as distinct hypothalamic nuclei during torpor in several species [29, 31, 62, 81, 82, 83, 84, 85]. It is unclear whether this increase results from specific mRNA stabilization, or whether these factors are still transcribed. However, the distinct spatial distribution at different stages during the torpor arousal cycle suggests that they contribute to maintaining functionality of specific hypothalamic nuclei relevant to the torpor arousal cycle.

The iodothyronine deiodinase type II (*Dio2*) was decreased in both torpor states. *Dio2* is an enzyme, converting T4 into bioactive T3. This finding is consistent with previous studies suggesting that decreased T3 concentrations in the hypothalamus are involved in torpor control [31, 36, 55, 86, 87]. The thioredoxin‐interacting protein (*Txnip*) was increased in both torpor states. *Txnip* is an important regulator of cellular glucose and fatty acid metabolism in cells and thereby controls fuel use [88]. Increase during torpor is consistent with previous studies in various species [31, 34, 89].

The somatostatin receptor type 2 (*Sstr2*), of the growth axis was decreased in early torpor (ET vs IBA). The relevance of somatostatin and its receptors for torpor behavior have previously been shown in Djungarian hamsters (*P. sungorus*) and European hamster (*C. cricetus*) [27, 29, 90, 91, 92].

The glucose transporter member 1 (*Glut1*) was increased in late torpor. In the hypothalamus of the 13‐lined ground squirrel (*I. tridecemlineatus*), *Glut1* was also increased in torpor entry, late torpor, and torpor arousal, each compared with IBA [31]. This indicates facilitated glucose transport to the brain during torpor. The neuropeptide Y receptor type 1 (*Npy1r*) was decreased in late torpor (LT vs IBA). Hypothalamic neuropeptide Y signaling triggers an orexigenic response that has been shown to induce torpor‐like hypothermia in the Djungarian hamster via *Npy1r* [93, 94]. Accordingly, NPY signaling of satiety might be modulated during torpor in the dormouse via *Npy1r* expression.

## Conclusion

In line with previous studies on other hibernating species, the hypothalamic transcriptome of the garden dormouse shows specific gene expression at different stages of a torpor arousal cycle. Our results reveal distinct remodeling of pathways during torpor, related to hemostasis, extracellular matrix organization and transport of small molecules, that become less supported during late torpor and may be related to the necessity for a reversal of metabolic functions as occurring during periodic arousal [14, 95, 96, 97, 98, 99].

## Conflict of interest

The authors declare no conflict of interest.

## Author contributions

AH and SG conceived the project and contributed equally. SG and GS performed the animal work. SG and AK‐H performed the sampling. EH and JB performed the bioinformatics, statistics, and data analyses. EH, JB, SG and AH interpreted the data. EH and AH wrote the manuscript, which was carefully revised by all authors. All authors agree to be accountable for the content of the work.

## Supporting information


**Fig. S1.** Transcript length.

**Fig. S2.** Hypothalamus specific gene expression.

**Table S1.** Background information animals.
**Table S2.** Number of read pairs.
**Table S3.** Background information of lab work.

**File S1.** Gene expression.

**File S2.** Pathways.

**File S3.** Indicator genes.

## Data Availability

Data that support the findings of this study are openly available in NCBI's Gene Expression Omnibus and are accessible through https://www.ncbi.nlm.nih.gov/geo/query/acc.cgi?acc=GSE207494, GEO Series accession number GSE207494. Supplementary materials are contained in the Supporting Information or available in the Open Access Repositorium of Ulm University OPARU at https://doi.org/10.18725/OPARU‐50321.

## References

[1] Ruf T and Geiser F (2015) Daily torpor and hibernation in birds and mammals. Biol Rev 90, 891–926. 25123049 10.1111/brv.12137 PMC4351926

[2] Geiser F (2021) Ecological Physiology of Daily Torpor and Hibernation. Springer International Publishing, Cham.

[3] Wilz M and Heldmaier G (2000) Comparison of hibernation, estivation and daily torpor in the edible dormouse, *Glis glis* . J Comp Physiol B 170, 511–521. 11128441 10.1007/s003600000129

[4] Dark J (2005) Annual lipid cycles in hibernators: integration of physiology and behavior. Annu Rev Nutr 25, 469–497. 16011475 10.1146/annurev.nutr.25.050304.092514

[5] Gil‐Delgado JA , Cabaret P , Declercq S , Gómez J and Sánchez I (2006) Winter reproduction of *Eliomys quercinus* (Rodentia) in the orange groves of Sagunto (Valencia, Spain). Mammalia 76–79.

[6] Geiser F (2020) Seasonal expression of avian and mammalian daily torpor and hibernation: not a simple summer‐winter affair. Front Physiol 11, 1–19. 32508673 10.3389/fphys.2020.00436 PMC7251182

[7] Scherbarth F and Steinlechner S (2010) Endocrine mechanisms of seasonal adaptation in small mammals: from early results to present understanding. J Comp Physiol B 180, 935–952. 20640428 10.1007/s00360-010-0498-2

[8] Storey KB and Storey JM (2005) Mammalian hibernation: biochemical adaptation and gene expression. In Functional Metabolism, pp. 443–471. John Wiley & Sons, Inc, Hoboken, NJ.

[9] Carey HV , Andrews MT and Martin SL (2003) Mammalian hibernation: cellular and molecular responses to depressed metabolism and low temperature. Physiol Rev 83, 1153–1181. 14506303 10.1152/physrev.00008.2003

[10] Müller D , Hauer J , Schöttner K , Fritzsche P and Weinert D (2015) Seasonal adaptation of dwarf hamsters (genus *Phodopus*): differences between species and their geographic origin. J Comp Physiol B 185, 917–930. 26323343 10.1007/s00360-015-0926-4

[11] Heldmaier G , Ortmann S and Elvert R (2004) Natural hypometabolism during hibernation and daily torpor in mammals. Respir Physiol Neurobiol 141, 317–329. 15288602 10.1016/j.resp.2004.03.014

[12] Hampton M , Nelson BT and Andrews MT (2010) Circulation and metabolic rates in a natural hibernator: an integrative physiological model. Am J Physiol Regul Integr Comp Physiol 299, 1478–1488. 10.1152/ajpregu.00273.2010 PMC3008751 20844258

[13] Buck CL and Barnes BM (2000) Effects of ambient temperature on metabolic rate, respiratory quotient, and torpor in an arctic hibernator. Am J Physiol Regul Integr Comp Physiol 279, R255–R262. 10896889 10.1152/ajpregu.2000.279.1.R255

[14] Ruf T , Gasch K , Stalder G , Gerritsmann H and Giroud S (2021) An hourglass mechanism controls torpor bout length in hibernating garden dormice. J Exp Biol 224, jeb243456. 34762135 10.1242/jeb.243456 PMC8714077

[15] van Breukelen F and Martin SL (2015) The hibernation continuum: physiological and molecular aspects of metabolic plasticity in mammals. Physiology 30, 273–281. 26136541 10.1152/physiol.00010.2015

[16] Giroud S , Habold C , Nespolo RF , Mejías C , Terrien J , Logan SM , Henning RH and Storey KB (2021) The torpid state: recent advances in metabolic adaptations and protective mechanisms. Front Physiol 11, 1–24. 10.3389/fphys.2020.623665 PMC7854925 33551846

[17] Mahlert B , Gerritsmann H , Stalder G , Ruf T , Zahariev A , Blanc S and Giroud S (2018) Implications of being born late in the active season for growth, fattening, torpor use, winter survival and fecundity. Elife 7, 1–25. 10.7554/eLife.31225 PMC5819945 29458712

[18] Nowack J , Tarmann I , Hoelzl F , Smith S , Giroud S and Ruf T (2019) Always a price to pay: hibernation at low temperatures comes with a trade‐off between energy savings and telomere damage. Biol Lett 15, 20190466. 31573426 10.1098/rsbl.2019.0466 PMC6832184

[19] Giroud S , Stalder G , Gerritsmann H , Kübber‐Heiss A , Kwak J , Arnold W and Ruf T (2018) Dietary lipids affect the onset of hibernation in the garden dormouse (*Eliomys quercinus*): implications for cardiac function. Front Physiol 9, 1–12. 30279661 10.3389/fphys.2018.01235 PMC6153335

[20] Arnold W , Giroud S , Valencak TG and Ruf T (2015) Ecophysiology of omega fatty acids: a lid for every jar. Physiology 30, 232–240. 25933823 10.1152/physiol.00047.2014

[21] Pajunen I (1983) Ambient temperature dependence of the body temperature and of the duration of the hibernation periods in the garden dormouse, *Eliomys quercinus* L. Cryobiology 20, 690–697. 6661917 10.1016/0011-2240(83)90073-1

[22] Daan S (1972) Periodicity of heterothermy in the garden dormouse, *Eliomys quercinus* (L.). Neth J Zool 23, 237–265.

[23] Lanni A , Martins R , Ambid L and Goglia F (1990) Liver and brown fat mitochondrial response to cold in the garden dormouse (*Eliomys quercinus*). Comp Biochem Physiol B 97, 809–813. 2085962 10.1016/0305-0491(90)90126-e

[24] Giroud S , Zahn S , Criscuolo F , Chery I , Blanc S , Turbill C and Ruf T (2014) Late‐born intermittently fasted juvenile garden dormice use torpor to grow and fatten prior to hibernation: consequences for ageing processes. Proc Biol Sci 281, 1–9. 10.1098/rspb.2014.1131 PMC4240977 25377448

[25] Heldmaier G and Ruf T (1992) Body temperature and metabolic rate during natural hypothermia in endotherms. J Comp Physiol B 162, 696–706. 1494028 10.1007/BF00301619

[26] Roh E , Song DK and Kim M‐S (2016) Emerging role of the brain in the homeostatic regulation of energy and glucose metabolism. Exp Mol Med 48, e216. 26964832 10.1038/emm.2016.4 PMC4892882

[27] Jastroch M , Giroud S , Barrett P , Geiser F , Heldmaier G and Herwig A (2016) Seasonal control of mammalian energy balance: recent advances in the understanding of daily torpor and hibernation. J Neuroendocrinol 28, 1–10. 10.1111/jne.12437 27755687

[28] Cubuk C , Kemmling J , Fabrizius A and Herwig A (2017) Transcriptome analysis of hypothalamic gene expression during daily torpor in Djungarian hamsters (*Phodopus sungorus*). Front Neurosci 11, 122. 28348515 10.3389/fnins.2017.00122 PMC5346580

[29] Haugg E , Borner J , Diedrich V and Herwig A (2021) Comparative transcriptomics of the Djungarian hamster hypothalamus during short photoperiod acclimation and spontaneous torpor. FEBS Open Bio 12, 443–459. 10.1002/2211-5463.13350 PMC8804604 34894101

[30] Gautier C , Bothorel B , Ciocca D , Valour D , Gaudeau A , Dupré C , Lizzo G , Brasseur C , Riest‐Fery I , Stephan JP *et al*. (2018) Gene expression profiling during hibernation in the European hamster. Sci Rep 8, 1–17. 30177816 10.1038/s41598-018-31506-2 PMC6120936

[31] Fu R , Gillen AE , Grabek KR , Riemondy KA , Epperson LE , Bustamante CD , Hesselberth JR and Martin SL (2021) Dynamic RNA regulation in the brain underlies physiological plasticity in a hibernating mammal. Front Physiol 11, 1–18. 10.3389/fphys.2020.624677 PMC7848201 33536943

[32] Schwartz C , Hampton M and Andrews MT (2013) Seasonal and regional differences in gene expression in the brain of a hibernating mammal. PLoS One 8, e58427. 23526982 10.1371/journal.pone.0058427 PMC3603966

[33] Schwartz C , Hampton M and Andrews MT (2015) Hypothalamic gene expression underlying pre‐hibernation satiety. Genes Brain Behav 14, 310–318. 25640202 10.1111/gbb.12199 PMC4386626

[34] Nespolo RF , Gaitan‐Espitia JD , Quintero‐Galvis JF , Fernandez FV , Silva AX , Molina C , Storey KB and Bozinovic F (2018) A functional transcriptomic analysis in the relict marsupial *Dromiciops gliroides* reveals adaptive regulation of protective functions during hibernation. Mol Ecol 27, 4489–4500. 30240506 10.1111/mec.14876

[35] Diedrich V , Kumstel S and Steinlechner S (2015) Spontaneous daily torpor and fasting‐induced torpor in Djungarian hamsters are characterized by distinct patterns of metabolic rate. J Comp Physiol B 185, 355–366. 25526676 10.1007/s00360-014-0882-4

[36] Cubuk C , Markowsky H and Herwig A (2017) Hypothalamic control systems show differential gene expression during spontaneous daily torpor and fasting‐induced torpor in the Djungarian hamster (*Phodopus sungorus*). PLoS One 12, e0186299. 29023516 10.1371/journal.pone.0186299 PMC5638525

[37] Melvin RG and Andrews MT (2009) Torpor induction in mammals: recent discoveries fueling new ideas. Trends Endocrinol Metab 20, 490–498. 19864159 10.1016/j.tem.2009.09.005 PMC2788021

[38] Logan SM and Storey KB (2017) Avoiding apoptosis during mammalian hibernation. Temperature 4, 15–17. 10.1080/23328940.2016.1211071 PMC5388434 28417098

[39] Huber N , Vetter S , Stalder G , Gerritsmann H and Giroud S (2021) Dynamic function and composition shift in circulating innate immune cells in hibernating garden dormice. Front Physiol 12, 1–12. 10.3389/fphys.2021.620614 PMC7970003 33746769

[40] Herwig A , Borner J and Haugg E (2023) Dataset: Bioinformatic scripts of the study: Haugg E, Borner J, Stalder G, Kübber‐Heiss A, Giroud S, Herwig A (submitted September 2023) Comparative transcriptomics of the hibernating garden dormouse hypothalamus. Open Access Repositorium of Ulm Universit. doi: 10.18725/OPARU-50321

[41] Herwig A , Haugg E and Giroud S (2022) Dataset: Comparative transcriptomics of the garden dormouse hypothalamus during early torpor, late torpor and interbout arousal of hibernation. Gene Expression Omnibus. https://www.ncbi.nlm.nih.gov/geo/query/acc.cgi?acc=GSE207494

[42] Grabherr MG , Haas BJ , Yassour M , Levin JZ , Thompson DA , Amit I , Adiconis X , Fan L , Raychowdhury R , Zeng Q *et al*. (2013) Trinity: reconstructing a full‐length transcriptome without a genome from RNA‐seq data. Nat Biotechnol 29, 644–652. 10.1038/nbt.1883 PMC3571712 21572440

[43] Haas BJ , Papanicolaou A , Yassour M , Grabherr M , Blood PD , Bowden J , Couger MB , Eccles D , Li B , Lieber M *et al*. (2013) *De novo* transcript sequence reconstruction from RNA‐seq using the trinity platform for reference generation and analysis. Nat Protoc 8, 1494–1512. 23845962 10.1038/nprot.2013.084 PMC3875132

[44] Genome Reference Consortium (2020) Dataset: Reference Proteome of *Mus musculus* (GRCm39). National Center for Biotechnology Information. https://www.ncbi.nlm.nih.gov/assembly/GCF_000001635.27/

[45] Genome Reference Consortium (2020) Dataset: Protein Table for *Mus musculus* (GRCm39). National Center for Biotechnology Information. https://www.ncbi.nlm.nih.gov/genome/browse#!/proteins/52/992563%7CMusmusculus

[46] Altschul SF , Gish W , Miller W , Myers EW and Lipman DJ (1990) Basic local alignment search tool. J Mol Biol 215, 403–410. 2231712 10.1016/S0022-2836(05)80360-2

[47] Langmead B (2010) Aligning short sequencing reads with Bowtie. Curr Protoc Bioinformatics Chapter 11, Unit 11.7. doi: 10.1002/0471250953.bi1107s32 PMC3010897 21154709

[48] Li B and Dewey CN (2011) RSEM: accurate transcript quantification from RNA‐seq data with or without a reference genome. BMC Bioinformatics 12, 323. 21816040 10.1186/1471-2105-12-323 PMC3163565

[49] Love MI , Huber W and Anders S (2014) Moderated estimation of fold change and dispersion for RNA‐seq data with DESeq2. Genome Biol 15, 1–21. 10.1186/s13059-014-0550-8 PMC4302049 25516281

[50] The Gene Ontology Consortium , Ashburner M , Ball CA , Blake JA , Botstein D , Butler H , Cherry JM , Davis AP , Dolinski K , Dwight SS *et al*. (2000) Gene ontology: tool for the unification of biology. Nat Genet 25, 25–29. 10802651 10.1038/75556 PMC3037419

[51] The Gene Ontology Consortium , Carbon S , Douglass E , Good BM , Unni DR , Harris NL , Mungall CJ , Basu S , Chisholm RL , Dodson RJ *et al*. (2021) The gene ontology resource: enriching a GOld mine. Nucleic Acids Res 49, D325–D334. 33290552 10.1093/nar/gkaa1113 PMC7779012

[52] Mi H , Muruganujan A , Ebert D , Huang X and Thomas PD (2019) PANTHER version 14: more genomes, a new PANTHER GO‐slim and improvements in enrichment analysis tools. Nucleic Acids Res 47, D419–D426. 30407594 10.1093/nar/gky1038 PMC6323939

[53] Hong G , Zhang W , Li H , Shen X and Guo Z (2013) Separate enrichment analysis of pathways for up‐ and downregulated genes. J R Soc Interface 11, 20130950. 24352673 10.1098/rsif.2013.0950 PMC3899863

[54] Gillespie M , Jassal B , Stephan R , Milacic M , Rothfels K , Senff‐Ribeiro A , Griss J , Sevilla C , Matthews L , Gong C *et al*. (2022) The reactome pathway knowledgebase 2022. Nucleic Acids Res 50, D687–D692. 34788843 10.1093/nar/gkab1028 PMC8689983

[55] Chmura HE , Duncan C , Saer B , Moore JT , Barnes BM , Loren Buck C , Christian HC , Loudon ASI and Williams CT (2022) Hypothalamic remodeling of thyroid hormone signaling during hibernation in the arctic ground squirrel. Commun Biol 5, 1–13. 35606540 10.1038/s42003-022-03431-8 PMC9126913

[56] Cubuk C , Bank JHH and Herwig A (2016) The chemistry of cold: mechanisms of torpor regulation in the Siberian hamster. Physiology 31, 51–59. 26674551 10.1152/physiol.00028.2015

[57] Van Breukelen F and Martin SL (2001) Translational initiation is uncoupled from elongation at 18°C during mammalian hibernation. Am J Physiol Regul Integr Comp Physiol 281, 1374–1379. 10.1152/ajpregu.2001.281.5.R1374 11641105

[58] Diaz MB , Lange M , Heldmaier G and Klingenspor M (2004) Depression of transcription and translation during daily torpor in the Djungarian hamster (*Phodopus sungorus*). J Comp Physiol B 174, 495–502. doi: 10.1007/s00360-004-0436-2 15232707

[59] van Breukelen F and Martin SL (2002) Reversible depression of transcription during hibernation. J Comp Physiol B 172, 355–361. doi: 10.1007/s00360-002-0256-1 12122451

[60] Frerichs KU , Smith CB , Brenner M , DeGracia DJ , Krause GS , Marrone L , Dever TE and Hallenbeck JM (1998) Suppression of protein synthesis in brain during hibernation involves inhibition of protein initiation and elongation. Proc Natl Acad Sci USA 95, 14511–14516. 9826731 10.1073/pnas.95.24.14511 PMC24404

[61] Grabek KR , Behn CD , Barsh GS , Hesselberth JR and Martin SL (2015) Enhanced stability and polyadenylation of select mRNAs support rapid thermogenesis in the brown fat of a hibernator. Elife 2015, 1–19. 10.7554/eLife.04517 PMC4383249 25626169

[62] Bratincsak A , McMullen D , Miyake S , Toth ZE , Hallenbeck JM and Palkovits M (2007) Spatial and temporal activation of brain regions in hibernation: c‐fos expression during the hibernation bout in thirtheen‐lined ground squirrel. J Comp Neurol 505, 443–458. 17912746 10.1002/cne.21507 PMC2774134

[63] Sonntag M and Arendt T (2019) Neuronal activity in the hibernating brain. Front Neuroanat 13, 71. 31338028 10.3389/fnana.2019.00071 PMC6629779

[64] Periayah MH , Halim AS and Saad AZM (2017) Mechanism action of platelets and crucial blood coagulation pathways in hemostasis. Int J Hematol Oncol Stem Cell Res 11, 319–327. 29340130 PMC5767294

[65] Hou Y , Carrim N , Wang Y , Gallant RC , Marshall A and Ni H (2015) Platelets in hemostasis and thrombosis: novel mechanisms of fibrinogen‐independent platelet aggregation and fibronectinmediated protein wave of hemostasis. J Biomed Res 29, 437–444. 26541706 10.7555/JBR.29.20150121 PMC4662204

[66] Versteeg HH , Heemskerk JWM , Levi M and Reitsma PH (2013) New fundamentals in hemostasis. Physiol Rev 93, 327–358. 23303912 10.1152/physrev.00016.2011

[67] Frerichs KU , Kennedy C , Sokoloff L and Hallenbeck JM (1994) Local cerebral blood flow during hibernation, a model of natural tolerance to “cerebral ischemia”. J Cereb Blood Flow Metab 14, 193–205. 8113316 10.1038/jcbfm.1994.26

[68] Lindell SL , Klahn SL , Piazza TM , Mangino MJ , Torrealba JR , Southard JH and Carey HV (2005) Natural resistance to liver cold ischemia–reperfusion injury associated with the hibernation phenotype. Am J Physiol Gastrointest Liver Physiol 288, 473–480. 10.1152/ajpgi.00223.2004 15701622

[69] Kurtz CC , Lindell SL , Mangino MJ and Carey HV (2006) Hibernation confers resistance to intestinal ischemia–reperfusion injury. Am J Physiol Gastrointest Liver Physiol 291, 895–901. 10.1152/ajpgi.00155.2006 16751173

[70] De Vrij EL , Vogelaar PC , Goris M , Houwertjes MC , Herwig A , Dugbartey GJ , Boerema AS , Strijkstra AM , Bouma HR and Henning RH (2014) Platelet dynamics during natural and pharmacologically induced torpor and forced hypothermia. PLoS One 9, 1–12. 10.1371/journal.pone.0093218 PMC3982955 24722364

[71] Cooper S , Sell S , Nelson L , Hawes J , Benrud JA , Kohlnhofer BM , Burmeister BR and Flood VH (2016) Von Willebrand factor is reversibly decreased during torpor in 13‐lined ground squirrels. J Comp Physiol B 186, 131–139. 26481634 10.1007/s00360-015-0941-5 PMC4838567

[72] Lau LW , Cua R , Keough MB , Haylock‐Jacobs S and Yong VW (2013) Pathophysiology of the brain extracellular matrix: a new target for remyelination. Nat Rev Neurosci 14, 722–729. 23985834 10.1038/nrn3550

[73] Schwartz MA (2001) Integrin signaling revisited. Trends Cell Biol 11, 466–470. 11719050 10.1016/s0962-8924(01)02152-3

[74] Ata R and Antonescu CN (2017) Integrins and cell metabolism: an intimate relationship impacting cancer. Int J Mol Sci 18, 189. 28106780 10.3390/ijms18010189 PMC5297821

[75] Larsen M , Artym VV , Green JA and Yamada KM (2006) The matrix reorganized: extracellular matrix remodeling and integrin signaling. Curr Opin Cell Biol 18, 463–471. 16919434 10.1016/j.ceb.2006.08.009

[76] Weidner KM , Sachs M and Birchmeier W (1993) The Met receptor tyrosine kinase transduces motility, proliferation, and morphogenic signals of scatter factor/hepatocyte growth factor in epithelial cells. J Cell Biol 121, 145–154. 8384622 10.1083/jcb.121.1.145 PMC2119778

[77] Beviglia L and Kramer RH (1999) HGF induces FAK activation and integrin‐mediated adhesion in MTLn3 breast carcinoma cells. Int J Cancer 83, 640–649. 10521801 10.1002/(sici)1097-0215(19991126)83:5<640::aid-ijc13>3.0.co;2-d

[78] Trusolino L , Bertotti A and Comoglio PM (2001) A signaling adapter function for α6β4 integrin in the control of HGF‐dependent invasive growth. Cell 107, 643–654. 11733063 10.1016/s0092-8674(01)00567-0

[79] Chen S‐Y and Chen H‐C (2006) Direct interaction of focal adhesion kinase (FAK) with Met is required for FAK to promote hepatocyte growth factor‐induced cell invasion. Mol Cell Biol 26, 5155–5167. 16782899 10.1128/MCB.02186-05 PMC1489146

[80] Hu C , Tao L , Cao X and Chen L (2020) The solute carrier transporters and the brain: physiological and pharmacological implications. Asian J Pharm Sci 15, 131–144. 32373195 10.1016/j.ajps.2019.09.002 PMC7193445

[81] Revel FG , Herwig A , Garidou M‐L , Dardente H , Menet JS , Masson‐Pévet M , Simonneaux V , Saboureau M and Pévet P (2007) The circadian clock stops ticking during deep hibernation in the European hamster. Proc Natl Acad Sci USA 104, 13816–13820. 17715068 10.1073/pnas.0704699104 PMC1959465

[82] Ikeno T , Williams CT , Buck CL , Barnes BM and Yan L (2017) Clock gene expression in the suprachiasmatic nucleus of hibernating Arctic ground squirrels. J Biol Rhythms 32, 246–256. 28452286 10.1177/0748730417702246

[83] O'Hara BF , Watson FL , Srere HK , Kumar H , Wiler SW , Welch SK , Bitting L , Heller HC and Kilduff TS (1999) Gene expression in the brain across the hibernation cycle. J Neurosci 19, 3781–3790. 10234010 10.1523/JNEUROSCI.19-10-03781.1999 PMC6782720

[84] Bitting L , Sutin EL , Watson FL , Leard LE , O'Hara BF , Craig Heller H and Kilduff TS (1994) C‐fos mRNA increases in the ground squirrel suprachiasmatic nucleus during arousal from hibernation. Neurosci Lett 165, 117–121. 8015710 10.1016/0304-3940(94)90723-4

[85] Yan J , Barnes BM , Kohl F and Marr TG (2008) Modulation of gene expression in hibernating arctic ground squirrels. Physiol Genomics 32, 170–181. 17925484 10.1152/physiolgenomics.00075.2007

[86] Murphy M , Jethwa PH , Warner A , Barrett P , Nilaweera KN , Brameld JM and Ebling FJP (2012) Effects of manipulating hypothalamic triiodothyronine concentrations on seasonal body weight and torpor cycles in Siberian hamsters. Endocrinology 153, 101–112. 22028444 10.1210/en.2011-1249

[87] Bank JHH , Cubuk C , Wilson D , Rijntjes E , Kemmling J , Markowsky H , Barrett P and Herwig A (2017) Gene expression analysis and microdialysis suggest hypothalamic triiodothyronine (T3) gates daily torpor in Djungarian hamsters (*Phodopus sungorus*). J Comp Physiol B 187, 857–868. 28365894 10.1007/s00360-017-1086-5

[88] Schwartz GJ and Blouet C (2011) Nutrient‐sensing hypothalamic TXNIP links nutrient excess to energy imbalance in mice. J Neurosci 31, 6019–6027. 21508227 10.1523/JNEUROSCI.6498-10.2011 PMC3100164

[89] Hand LE , Saer BRC , Hui ST , Jinnah HA , Steinlechner S , Loudon ASI and Bechtold DA (2013) Induction of the metabolic regulator txnip in fasting‐induced and natural torpor. Endocrinology 154, 2081–2091. 23584857 10.1210/en.2012-2051 PMC3740491

[90] Nürnberger F , Pleschka K , Masson‐Pévet M and Pévet P (1997) The somatostatin system of the brain and hibernation in the European hamster (*Cricetus cricetus*). Cell Tissue Res 288, 441–447. 9134858 10.1007/s004410050831

[91] Dumbell RA , Scherbarth F , Diedrich V , Schmid HA , Steinlechner S and Barrett P (2015) Somatostatin agonist pasireotide promotes a physiological state resembling short‐day acclimation in the photoperiodic male Siberian hamster (*Phodopus sungorus*). J Neuroendocrinol 27, 588–599. 25950084 10.1111/jne.12289

[92] Scherbarth F , Diedrich V , Dumbell RA , Schmid HA , Steinlechner S and Barrett P (2015) Somatostatin receptor activation is involved in the control of daily torpor in a seasonal mammal. Am J Physiol Regul Integr Comp Physiol 309, R668–R674. 26157058 10.1152/ajpregu.00191.2015

[93] Paul MJ , Freeman DA , Jin HP and Dark J (2005) Neuropeptide Y induces torpor‐like hypothermia in Siberian hamsters. Brain Res 1055, 83–92. 16098953 10.1016/j.brainres.2005.06.090

[94] Dark J and Pelz KM (2008) NPY Y1 receptor antagonist prevents NPY‐induced torporlike hypothermia in cold‐acclimated Siberian hamsters. Am J Physiol Regul Integr Comp Physiol 294, 236–245. 10.1152/ajpregu.00587.2007 17989140

[95] Prendergast BJ , Freeman DA , Zucker I and Nelson RJ (2002) Periodic arousal from hibernation is necessary for initiation of immune responses in ground squirrels. Am J Physiol Regul Integr Comp Physiol 282, 1054–1062. 10.1152/ajpregu.00562.2001 11893609

[96] Ruf T and Arnold W (2008) Effects of polyunsaturated fatty acids on hibernation and torpor: a review and hypothesis. Am J Physiol Regul Integr Comp Physiol 294, R1044–R1052. 18171691 10.1152/ajpregu.00688.2007

[97] Epperson LE , Karimpour‐Fard A , Hunter LE and Martin SL (2011) Metabolic cycles in a circannual hibernator. Physiol Genomics 43, 799–807. 21540299 10.1152/physiolgenomics.00028.2011 PMC3132838

[98] Humphries MM , Thomas DW and Kramer DL (2003) The role of energy availability in mammalian hibernation: a cost–benefit approach. Physiol Biochem Zool 76, 165–179. 12794670 10.1086/367950

[99] Strijkstra A and Daan S (2003) Sleep during arousal episodes as a function of prior torpor duration in hibernating European ground squirrels. J Sleep Res 6, 36–43. 10.1046/j.1365-2869.1997.00024.x 9125697

