# Supplementary material for: Comparative transcriptomics of the garden dormouse hypothalamus during hibernation
Source: FEBS Open Bio. 2023 Dec 18;14(2):241–57. doi: 10.1002/2211-5463.13731 (PMC10839406; doi:10.1002/2211-5463.13731)
Supplement: Supplementary file 1 — Fig. S1. Transcript length. [file FEB4-14-241-s004.pdf]

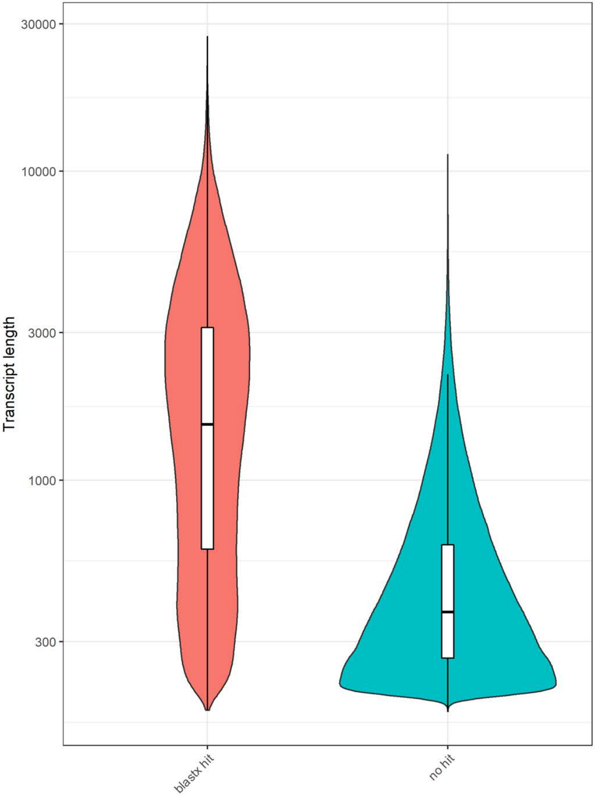

Suppl. Figure 1: Transcript lengths. Distribution of transcript lengths for transcripts that had a significant blastx hit against the mouse proteome and those that did not. The width of each violin indicates the number of transcripts at the respective size range. The boxes show the median transcript length (center line) and the 25th and 75th percentiles (upper and lower edges of the boxes, respectively).
