# Supplementary material for: Comparative transcriptomics of the garden dormouse hypothalamus during hibernation
Source: FEBS Open Bio. 2023 Dec 18;14(2):241–57. doi: 10.1002/2211-5463.13731 (PMC10839406; doi:10.1002/2211-5463.13731)
Supplement: Supplementary file 2 — Fig. S2. Hypothalamus specific gene expression. [file FEB4-14-241-s001.pdf]

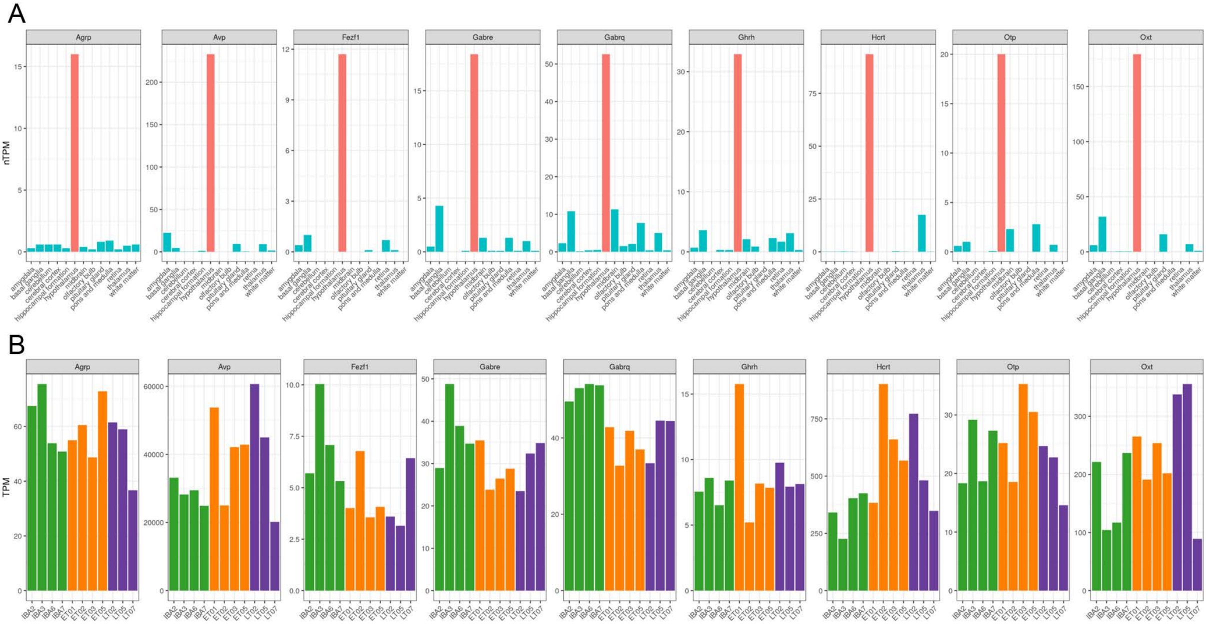


Suppl. Figure 2: Hypothalamus specific gene expression. A. Normalized mouse expression data from the Human Protein Atlas for 13 brain regions in the mouse of 9 hypothalamus specific genes. B. The same 9 hypothalamus specific genes in all individual dormouse samples.
