# Supplementary material for: Comparative transcriptomics of the garden dormouse hypothalamus during hibernation
Source: FEBS Open Bio. 2023 Dec 18;14(2):241–57. doi: 10.1002/2211-5463.13731 (PMC10839406; doi:10.1002/2211-5463.13731)
Supplement: Supplementary file 3 — Table S1. Background information animals. Table S2. Number of read pairs. Table S3. Background information of lab work. [file FEB4-14-241-s005.pdf]

Supplementary File 1

Supplementary Table 1: Background informations of animals. All eleven animals were adults kept at constant darkness without food and water at 5 °C to enable hibernation. Each animal is identified by the sampling group and a running number. The sex is given for females (f) and males (m). The duration is defined for torpor as time between Tb < 18 °C and sacrifice, and for IBA as time between Tb > 18 °C and sacrifice. Mean value with standard deviation of the mean (Std. Dev.) is given for each animal and group as early torpor (ET), late torpor (LT), torpor (ET+LT), interbout arousal (IBA). The hypothalamic sample of dormouse LT01 (sex: female, terminal Tb: 4.09 °C, terminal body mass: 77.4 g, sacrificed on 09.02.2015 in late torpor after 222.9 hours with Tb < 18 °C) was excluded from the analyses due to an undetectable RIN.

group	animal ID	sex	terminal Tb [°C]	terminal body mass [g]	date of sacrifice	duration [hours]
ET	ET01	m	5.31	86.4	24.02.2015	25.0
	ET02	f	4.24	84.0	03.02.2015	40.6
	ET03	m	5.08	92.7	04.02.2015	40.9
	ET05	m	4.40	87.9	04.02.2015	38.9
LT	LT02	m	5.54	116.7	03.02.2015	229.2
	LT05	m	5.14	98.1	13.02.2015	227.6
	LT07	f	4.62	95.6	12.02.2015	234.0
IBA	IBA2	f	37.03	74.5	22.03.2015	-
	IBA3	f	37.02	87.6	22.03.2015	3.1
	IBA6	m	36.86	90.9	19.02.2015	2.4
	IBA7	f	36.17	86.0	05.03.2015	5.8
ET (n = 4)	mean	-	4.8	87.8	-	36.3
	Std. Dev.	-	0.5	3.7	-	7.6
LT (n = 3)	mean	-	5.1	103.5	-	230.3
	Std. Dev.	-	0.5	11.5	-	3.3
ET+LT (n = 7)	mean		4.9	94.5	-	-
	Std. Dev.		0.5	11.0	-	-
IBA (n = 4)	mean	-	36.8	84.8	-	3.7
	Std. Dev.	-	0.4	7.1	-	1.8
all (n = 11)	mean	-	-	90.9	-	-
	Std. Dev.	-	-	10.6	-	-

Supplementary Table 2: Number of read pairs. Each animal is identified by the sampling group (early torpor – ET, late torpor – LT, interbout arousal – IBA) and a running number. mRNA-Seq was conducted on an Illumina NextSeq 500 (paired end, 4 lanes L001 to L004). The hypothalamic sample of dormouse LT01 was excluded from the analyses due to an undetectable RIN.

	group	sample ID	read pairs					
			L001	L002	L003	L004	average	sum
	ET	ET01	8,616,141	8,534,720	8,752,618	8,515,629	8,604,777	34,419,108
		ET02	7,278,826	7,180,590	7,386,325	7,147,440	7,248,295	28,993,181
		ET03	9,473,916	9,425,198	9,614,613	9,404,048	9,479,444	37,917,775
		ET05	7,808,047	7,719,751	7,923,083	7,708,887	7,789,942	31,159,768
	LT	LT01	9,438,251	9,380,250	9,590,752	9,362,964	9,443,054	37,772,217
		LT02	9,357,318	9,255,517	9,493,363	9,206,111	9,328,077	37,312,309
		LT05	8,598,337	8,495,974	8,723,407	8,484,806	8,575,631	34,302,524
		LT07	8,884,916	8,790,218	9,014,207	8,785,731	8,868,768	35,475,072
	IBA	IBA2	6,518,339	6,480,531	6,635,649	6,437,665	6,518,046	26,072,184
		IBA3	6,768,058	6,710,868	6,869,421	6,693,708	6,760,514	27,042,055
		IBA6	8,128,165	8,072,995	8,261,775	8,046,020	8,127,239	32,508,955
		IBA7	8,050,117	7,984,719	8,173,036	7,960,485	8,042,089	32,168,357
**min**			6,437,665				-	26,072,184
**average**			8,232,156				8,232,156	32,928,625
**max**			9,614,613				-	37,917,775
**sum**			-				-	**395,143,505**

Supplementary Table 3: Background information of lab work. Volume was orientated on tissue weight. Total RNA was isolated using peqGOLD TrifastTM (Peqlab, Erlangen, Germany) and purified using the Crystal RNA MiniKit (Biolabproducts, Bebensee, Germany) including an on-column digestion with RNase-free DNase (Quiagen, Hilden, Germany). RNA purity was assessed by the 260/280 nm ratio on a NanoDrop 1000 spectrophotometer. RNA integrity was proven by formaldehyde agarose gel electrophoresis. The hypothalamic sample of dormouse LT01 was excluded from the analyses due to an undetectable RIN.

group	animal ID	volume [ml]	quantity [µg]	concentration [ng / µl]	quality [RIN]
ET	ET01	3	1.22	407	6.2
	ET02	3	1.29	429	6.4
	ET03	3	1.12	375	6.4
	ET05	2	1.32	662	6.3
LT	LT02	5	1.05	210	6.3
	LT05	2	1.18	590	6.5
	LT07	2	1.33	667	7.3
IBA	IBA2	3	1.36	452	6.5
	IBA3	3	1.03	345	7.2
	IBA6	3	0.99	329	6.7
	IBA7	3	1.31	436	6.5
all (n = 11)	min	2	0.99	210	6.2
	max	5	1.36	667	7.3
	mean	3	1.20	446	6.6
	Std. Dev.	1	0.13	143	0.4
